# Supplementary material for: Living kidney donation in a developing country
Source: PLoS One. 2022 May 10;17(5):e0268183. doi: 10.1371/journal.pone.0268183 (PMC9089923; doi:10.1371/journal.pone.0268183)
Supplement: S1 Data — (PDF) [file pone.0268183.s001.pdf]

Database key

Degree of relation: Unrelated = 0, First = 1, Second = 2

RLD/NRLD: RLD = 1, NRLD = 0

Sex: Male = 0, Female = 1

Ethnicity: Black African = 0, Caucasian = 1, Mixed = 2, Asian = 3

BMI: obese = 0, non-obese = 1

HIV status: HIV negative = 0, HIV positive = 1

Workup outcome: Accepted = 0, Rejected = 1, Recipient excluded = 2

ABO compatibility: ABO compatible = 0, ABO incompatible = 1

HLA crossmatch: Positive = 0, Negative = 1
